# The Modulatory Role of Vitamin C in Boldenone Undecylenate Induced Testicular Oxidative Damage and Androgen Receptor Dysregulation in Adult Male Rats

**DOI:** 10.3390/antiox9111053

**Published:** 2020-10-28

**Authors:** Amany Behairy, Nabela I. El-Sharkawy, Taghred M. Saber, Mohamed Mohamed Soliman, Mohamed M.M. Metwally, Ghada I. Abd El-Rahman, Yasmina M. Abd-Elhakim, Maha M. El Deib

**Affiliations:** 1 Department of Physiology, Faculty of Veterinary Medicine, Zagazig University, Zagazig 44511, Egypt; amanybehairy25688@gmail.com; 2 Department of Forensic Medicine and Toxicology, Faculty of Veterinary Medicine, Zagazig University, Zagazig 44511, Egypt; nabelaimam@hotmail.com (N.I.E.-S.); taghredsaber1982@gmail.com (T.M.S.); 3 Clinical Laboratory Sciences Department, Turabah University College, Taif University, P.O. Box 11099, Taif 21944, Saudi Arabia; mmsoliman@tu.edu.sa; 4 Biochemistry Department, Faculty of Veterinary Medicine, Benha University, Benha 13736, Egypt; 5 Department of Pathology, Faculty of Veterinary Medicine, Zagazig University, Zagazig 44511, Egypt; metywally@gmail.com; 6 Department of Clinical Pathology, Faculty of Veterinary Medicine, Zagazig University, Zagazig 44511, Egypt; gana660@gmail.com; 7 Department of Biochemistry, Faculty of Veterinary Medicine, Zagazig University, Zagazig 44511,Egypt; mahaeldeib@zu.edu.eg

**Keywords:** Vitamin C, boldenone undecylenate, oxidative stress, male fertility, testicular dysfunction, androgen receptor

## Abstract

Background: This study explored the effect of vitamin C (Vit-C) administration on the reproductive function of adult male Wistar rats injected with boldenone undecylenate (BOL). Methods: Rats were randomly assigned into control, vehicle control, Vit-C (120 mg/kg b.wt./day, orally), BOL (received 5 mg/kg b.wt./week, IM) and BOL+Vit-C-treated groups. After eight weeks, hormonal assay, semen evaluation, testicular enzymes, and antioxidants biomarkers were assessed. Besides, the histopathological and immunohistochemical investigations of the androgen receptor (AR) expression were performed. Results: The results revealed that serum testosterone, acid phosphatase, sorbitol dehydrogenase, sperm abnormalities, and testicular malondialdehyde were significantly incremented in the BOL-treated group. Testicular weight, sperm count, and sperm motility together with serum levels of luteinizing hormone, follicle-stimulating hormone, and estradiol, and testicular testosterone, catalase, superoxide dismutase, and reduced glutathione showed a significant decrease following BOL treatment. Besides, the AR immunoreactivity was significantly decreased in testicular tissues. Vit-C co-administration with BOL significantly relieved the BOL-induced sperm abnormalities, reduced sperm motility, testicular enzyme leakage, and oxidative damage. However, Vit-C could rescue neither BOL-induced hormonal disturbances nor AR down-regulation. Conclusions: The results provide further insight into the mechanisms of BOL-induced reproductive dysfunction and its partial recovery by Vit-C.

## 1. Introduction

The most potent androgen in the human body is testosterone (TES). Androgens’ effects are most apparent during puberty when they trigger profound physiological changes in the male body: secondary male properties production, the pattern of hair growth, sebaceous glands function, libido, and sperm maturation [1]. TES has multiple probable metabolic pathways [2]. Firstly, to exert an effect, it binds to the androgen receptor (AR) of target tissues. Secondly, it could be reduced to 5 α- dihydrotestosterone, which also targeted AR. Next to diverse pathways, TES may be aromatized to estradiol (E2) to exert estrogenic effects, typically water retention, breast tissue growth, and an increase in body fat deposition [3].

The anabolic-androgenic steroids (AAS) are synthetic TES derivatives modified to enhance the anabolic rather than the androgenic actions [4]. The prevalence of AAS ever in men is potentially 1% to 5% globally in the general population. It is much more prevalent in men than in women (<50:1), and long-term AAS users are mostly men [5]. AAS has relatively small molecules and can spread passively to cells in different tissues [6]. No tissues lack androgen (AR) receptors, which are part of the nuclear receptor super-family and are related to numerous AAS receptors. AAS exerts many anabolic complementary effects through pathways, including a psychoactive brain effect, glucocorticoid antagonism, and growth hormone development stimulation [7]. Over 100 TES synthetic derivatives were produced. They are well absorbed in the intestinal tract, then biotransformed during the first passing hepatic metabolism and partially bile-faced to the faces. They have different biological characteristics, as they are possible targets of aromatization and reduction [8]. Despite the therapeutic benefits of AAS because of their structural and functional resemblance to TES, competitive and aesthetic motivations often force users to exceed the recommended dose by upwards of 100 times to increase muscle size and strength and improve athletic performance [9].

Boldenone (BOL; 1,4-androstadiene-17b-ol-3-one) is a well-known androgenic steroid under the trade names Equipoise, Ganabol, Equigan, and Ultragan [10]. It is used by bodybuilders and athletes to develop their physical performance and muscle size by elevating positive nitrogen balance via motivating protein anabolism and decreasing protein catabolism besides the water, nitrogen, and electrolytes retention [11]. Also, BOL used as a growth promoter to improve the food conversion and growth in fattening animals [12] to accomplish more competent production of meat illegally [13].

The cardiovascular system, arrhythmia, erythrocytosis, hypertension, and ventricular dysfunction are the most significant effects of AAS’s long-term use [1]. Besides, hepatic, kidney, and endocrine systems have been reported [14]. Also, TES synthesis products trigger negative feedback on the hypothalamic-pituitary axis and thus inhibit both the follicle-stimulated hormone (FSH) and the luteinized hormone (LH) secretion. Infertility following an AAS abuse is commonly seen in sperm motility and morphological disorders associated with oligozoospermia or azoospermia [15]. Even after long retirements from AASs, some men with low TES levels may have hypogonadism that was not identified until AAS use began [16]. The commonly reported side effects by AAS users are fluid retention, testicular atrophy, gynecomastia, acne, and alopecia [17]. Also, in normal males, TES are aromatized to E2 with its known effects for normal libido and overall sexual activity but unfortunately, several AAS are not aromatized to E2 [18]. Thus, twenty-five percent of AAS users reported sexual dysfunction and androgen deficiency signs, including depression and fatigue [19].

Previous studies also show that AAS increase the levels of reactive oxygen species (ROS) and decrease the antioxidant capacity in several organs, such as the liver and the kidney [20,21]. The close link between increased ROS levels and the negative effect on sperm morphology has been previously defined, and the antioxidant status of semen, in particular, has been considerably lower than normal semen. [22]. Seminal oxidative stress negatively correlates with sperm count, function, and motility, adversely affecting fusion consequences needed for successful fertilization [23].

In this era, a growing interest is directed towards the use of natural supplements as reproprotecive therapies [24,25]. Vitamin C (Vit-C) is a water-soluble antioxidant ten times higher than that present in blood serum in seminal plasma [26]. Vit-C plays a key role in the fertility of men and animals as it safeguards spermatogenesis, retains the viability of sperms, prevents sperm agglutination, and raises the TES in serum [27]. Vit-C supplementation has also been shown to increase total sperm output and sperm concentration [28]. Vit-C seems to positively affect testicular and erectile functions in unhealthy and healthy persons with reproductive complications [29]. Some evidence suggests that testicular and erectile dysfunction is linked with low Vit-C levels in plasma [30]. In context, in infertile men with asthenozoospermia, seminal fluid had lower Vit-C levels and greater ROS values than those of fertile ones [31].

Based on the earlier pharmacological activities of Vit-C, especially the antioxidant and reprotective activity, we hypothesized that this natural product could counteract the impaired fertility associated with BOL administration in males. To test this hypothesis, semen evaluation, hormonal assay, testicular enzymes, and antioxidants biomarkers were assessed in adult male Wistar rats administered BOL and/or Vit-C for eight weeks. Also, histopathological and immunohistochemical investigations of the androgen receptor (AR) expression were performed to explore the underlying mechanisms.

## 2. Materials and Methods

### 2.1. Chemicals

BOL was purchased in the commercial form Equi-gan^®^ vial (Laboratorios Tornel Co., S.A., Mexico). Each vial contains an oily solution (50 mg BOL/mL vehicle). Vit-C was purchased in tablets, each containing 500 mg of ascorbic acid (Kahira Pharma Co., Cairo, Egypt). All other reagents and chemicals used were purchased from Sigma- Aldrich Co. (St.Louis, MO, USA) and were of analytical grade.

### 2.2. Animals

Forty adult male Wistar rats weighing 166.34 ± 2.32 g and 10–12 weeks of age were included in the current research. The animals were obtained from the laboratory animal house at the Faculty of Veterinary Medicine, Zagazig University. Rats were held in metal cages with a 40–60% relative humidity, 23 ± 2 °C temperature, and a 12-h light period. Rats have been fed a rodent diet in the experimental era, and water has been supplied *ad libitum*. Animals were adapted for two weeks to the experimental site. The Institutional Animal Care and Use Committee of Zagazig University approved the present protocol (ZU-IACUC/2/F/2020).

### 2.3. Experimental Design

Randomly, the animals were divided into five similar groups (8 per group).

- Group I (control group) orally given physiological saline.
- Group II (vehicle control group) was intramuscularly injected with 0.25 mL/kg b.wt. sesame oil as a vehicle once a week for eight weeks.
- Group III (Vit-C-treated group) was gavaged with Vit-C dissolved in distilled water (120 mg/kg b.wt.) every day for eight weeks [32].
- Group IV (BOL-treated group) was intramuscularly injected with BOL (5 mg/kg b.wt.) once a week for eight weeks, according to a previously mentioned dose of Bueno, et al. [33].
- Group V (BOL+Vit-C-treated group) was co-treated with BOL (5 mg/kg b.wt.) and Vit-C (120 mg/kg b.wt.) at the same declared duration and route.

### 2.4. Sampling

At the end of the experiment (after eight weeks), twenty-four hours next to the last administration, rats were weighed then euthanized under anesthesia. Blood samples were obtained from all rats in the different experimental groups in test tubes with no ethylenediaminetetraacetic acid left to coagulate for 20 min at room temperature, and centrifuged at 3000 rpm for 20 min for serum collection. The resultant serum was preserved at −20 °C until the hormonal analysis, including TES, LH, FSH, and E2 and testicular enzymes (acid phosphatase (ACP) and sorbitol dehydrogenase (SDH)).

Immediately after euthanasia, testes were carefully dissected out and weighed. The gondosomatic index has been calculated as (the testis weight/final body weight) ×100. In the cold phosphate-buffered saline (PBS, 0.01 mol/L, pH 7), the right testis for every rat was homogenized using the glass homogenizer. The resulting homogenates were centrifuged for 5 min at 5000× *g*; the supernatants were filtered with a Millipore philter (0.45 μm) to eliminate TES’s tissue debris hormone evaluation and oxidative stress assessment. In histopathological and immunohistochemical staining, the left one was fixed in 10% neutral buffered neutral formalin.

### 2.5. Semen Evaluation

The cauda epididymis of all rats testis was extracted for the sperm collection and transmitted into a sterilized Petri dish containing 2 mL of normal saline (37 °C). A small opening was then carried out with sterilized scissors to facilitate the sperm passing from the epididymis to achieve a spermiogram analysis of the epididymal material suspension. At 400× magnification, sperm motility percent was microscopically determined by the described protocol of Slott et al. [34]. Meanwhile, sperm cell concentration/milliliter semen was performed consistently with the method of Robb et al. [35]. For every sample, the count was repeated five times to reduce the error. Abnormalities of sperms have been reported using the Filler [36] protocol. Five hundred spermatozoa were observed per animal to evaluate the abnormalities frequency in the tail, neck/mid-piece, and head.

### 2.6. Hormonal Assay

Testosterone, LH, FSH, and E2 were determined via rat-specific enzyme-linked immunosorbent assay (ELISA) commercial kits of Elabscience^®^ Biotechnology Inc. (Cat. No.: MBS282195, MBS764675, MBS2502190, and MBS263466, respectively, Houston, TX, USA) with the Zirkin and Chen [37] method.

### 2.7. Testicular Enzymes Evaluation

ELISA kits (MBS046840) following the manufacturer’s instructions (MyBioSource, San Diego, CA, USA) was used to estimate serum acid phosphatase (ACP). Serum sorbitol dehydrogenase (SDH) was measured using specific ELISA kits for rats of Elabscience^®^ Biotechnology Inc. of Cat No. MBS166115, following the instruction at the enclosed pamphlets of the manufacturers.

### 2.8. Testicular Oxidative/Antioxidant Status

In testes homogenate, the catalase (CAT), superoxide dismutase (SOD), and reduced glutathione (GSH) levels were estimated by using kits reagents of Bio-diagnostic Co., Egypt according to the methods of Sinha [38], Nishikimi et al. [39], and Beutler et al. [40] respectively. Malondialdehyde (MDA) concentration was determined via the colorimetric assay of Ohkawa et al. [41].

### 2.9. Histopathological Evaluation

At the end of the experiment, the left testis from all animals was harvested according to standardized necropsy procedures [42], instantly fixed in 10% neutral buffered formalin for 48 h, dehydrated in ethanol (70–100%), cleared in two changes of xylene (one hour each), processed to paraffin impregnation and embedding, sectioned at five-microns tissue thickness, and stained with hematoxylin and eosin [43]. The slides were examined microscopically, and any histopathological alterations were reported. For morphometric analysis and quantitative lesion assessment of the testicular tissues, a representative one slide/animal was chosen. Five 10× and five 40× round or nearly round nonoverlapped randomly selected testicular images/slides were captured using AmScope digital camera attached to an Olympus light microscope. Subsequently, the frequencies of vascular congestions, inflammatory cell infiltrates, interstitial edema, diameters of STs, and the numbers of STs/images were quantified in the 10× images. However, the numbers of spermatids, spermatocytes, and spermatogonia/STs, the heights of germinal epithelium/STs, the numbers of STs manifested epithelial cell depletion, vacuolation, desquamation, necrosis, redundant basement membrane, spermatid retention, and giant cell formation/image were quantified in the 40× images. The measurements were performed via AmScope ToupView V3.7.13522 software, AmScope, United States, and the differentiation between germinal epithelial cells depended on the nuclear shape, size, and chromatin pattern. The results were expressed as percentages (means ± SE).

### 2.10. Immunohistochemical Investigation of Androgen Receptor Expression in the Sertoli and Leydig Cells

Consecutive five-micron thick testicular sections (one section/animal) were prepared from the formalin-fixed paraffin-embedded blocks, then stained for androgen receptor using rabbit monoclonal anti-androgen receptor antibody [ER179(2)]-ChIP Grade (ab108341) primary antibody, goat anti-rabbit IgG H&L (HRP) (ab205718) secondary antibody and 3,3′-Diaminobenzidine chromogen (Abcam, UK), according to the avidin-biotin-peroxidase complex protocol of Hsu, et al. [44]. For quantitative assessment of the androgen receptor expression, randomly selected five, non-duplicated 40× microscopic fields/animal were taken, and the AR immunoexpression was recorded by counting the numbers of positive Sertoli and Leydig cells/image.

### 2.11. Statistical Analysis

For each group, the data is shown as mean ± SE. Differences between groups were evaluated statistically through a one-way variance test (ANOVA), followed by a Duncan Post-Hoc test for comparison in pairs. Significant differences at *p* < 0.05 were considered. The computer program Graphpad (ISI Software, Philadelphia, PA, USA) was used for regression analysis and data collection.

## 3. Results

### 3.1. Effect of BOL and Vit-C on the Body and Reproductive Organ Weights

Table 1 displayed the variations in final body weight, weight gain, and gonadosomatic index in rats received BOL and/or Vit-C. The final body weight showed significant (*p* < 0.001) increases in Vit-C, BOL, and BOL+Vit-C treated groups compared to control and vehicle control groups. However, the body weight gain was significantly (*p* < 0.001) increased in BOL, and BOL+Vit-C treated groups with maximum increment in the BOL+Vit-C group. Meanwhile, the gonadosomatic index showed a significant (*p* < 0.001) reduction in rats injected with BOL alone and those co-treated with BOL and Vit-C compared to other experimental groups.

### 3.2. Effect of BOL and Vit-C on Spermiogram

**Figure 1:**
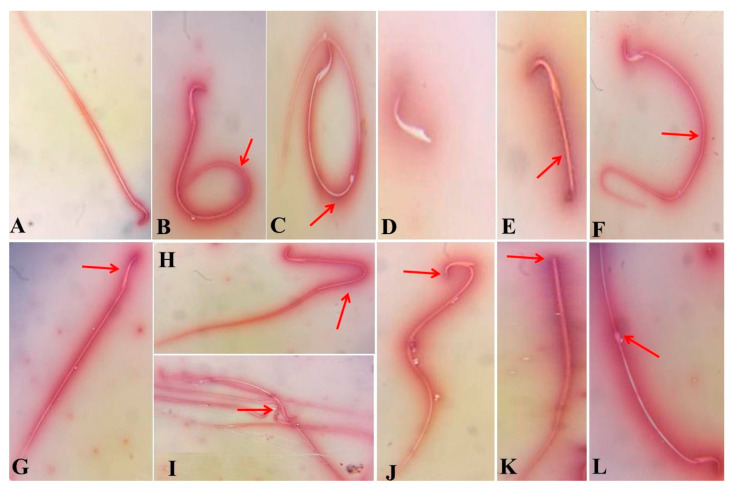
Photomicrographs of semen smears of the control, vehicle control, Vit-C, and BOL+ Vit-C treated rats showing sperms with normal morphology (**A**). Photomicrographs of eosin–nigrosin-stained semen smears of boldenone (BOL) and/or vitamin C (Vit-C) exposed rats for eight weeks showing different forms of epididymis sperm abnormalities in BOL tread rats, including coiled tail (**B**), looped tail (**C**), detached tail (**D**), short tail (**E**), curved tail (**F**), less hock head (**G**), Bent tail (**H**), Fused sperm (**I**), broken head (**J**), detached head (**K**), and protoplasmic droplet (**L**) (100×).

As presented in Table 1, intramuscular BOL injection once weekly for eight weeks resulted in a significant rise (*p* < 0.001) in the sperm abnormalities percent but reduced (*p* < 0.001) the motility percent and sperm count. These morphological alterations comprise short tail, detached tail, looped tail, coiled tail, curved tail, broken head, and detached head, as revealed in Figure 1 In contrast, the oral Vit-C dosing to adult male rats injected with BOL every day for eight weeks induced a significant (*p* < 0.001) decline in the sperm abnormalities percent compared with the BOL-only treated rats.

### 3.3. Effect of BOL and Vit-C on Male Reproductive Hormones

Table 2 exhibited the variations in total serum TES, LH, FSH, and E2 hormone together with testicular TES levels in the experimental groups under study. There was a significant increase (*p* < 0.001) in serum TES while a significant decrease in testicular TES in both BOL and BOL+Vit-C treated groups compared to control groups. Nevertheless, serum levels of LH, FSH, and E2 hormones were significantly (*p* < 0.001) decreased in both BOL and BOL+Vit-C treated rats compared to control ones. No significant changes in hormonal profiles were recorded between the control and Vit-C treated group.

### 3.4. Effect of BOL and Vit-C on Testicular Enzymes

As presented in Table 2, significant increments (*p* < 0.001) in the serum ACP and SDH were recorded in the BOL treated group compared to the control groups. BOL’s co-treatment with Vit-C elicits a significant decline of the ACP and SDH increment relative to the BOL alone treated group. No significant change was noted between the control group and Vit-C group in the ACP and SDH levels.

### 3.5. Effect of BOL and Vit-C on Testicular Antioxidant/Oxidative Status

**Figure 2:**
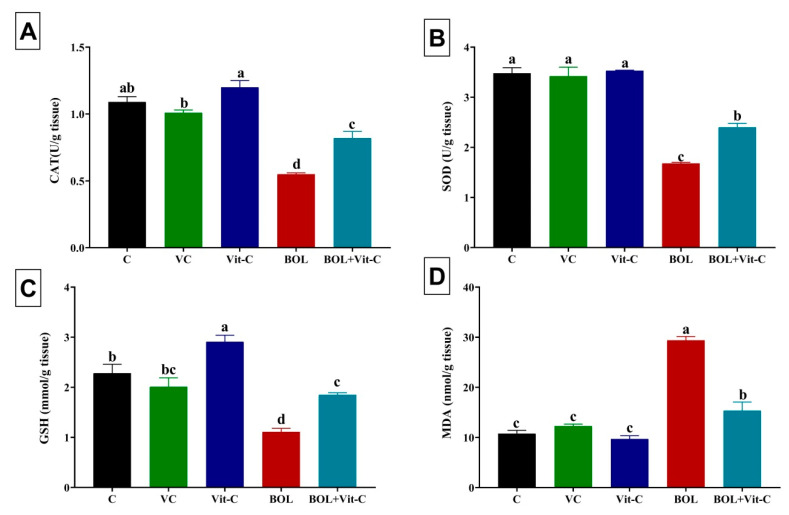
Changes in oxidative stress and lipid peroxidation indicators in boldenone (BOL) (5 mg/kg bwt/once a week, 8 weeks) and/or vitamin C (Vit-C) (120 mg/kg b.wt/daily, 8 weeks) treated adult male Wister rats. (**A**) Catalase, CAT; (**B**) Superoxide dismutase, SOD; (**C**) Reduced glutathione, GSH; and (**D**) Malondialadehyde, MDA. Data are expressed as the mean ± SE (*n* = 8 replicates). Columns carrying different superscripts (a, b, c, and d) are significantly different (One-way ANOVA) (*p* < 0.001).

As shown in Figure 2, daily oral Vit-C administration (120 mg/kg b.wt. for eight weeks) to adult male rats significantly (*p* < 0.001) augmented the levels of CAT and GSH in the testicular tissues relative control rats. While intramuscular BOL administration (5 mg/kg b.wt) once weekly for eight weeks caused a significant (*p* < 0.001) reduction in the testicular levels of CAT, SOD, and GSH but a significant rise in the testicular MDA concentration. Interestingly, the testicular tissue antioxidant status of BOL-treated rats was significantly (*p* < 0.001) improved by concurrent treatment with Vit-C.

### 3.6. Histopathological Evaluation

**Figure 3:**
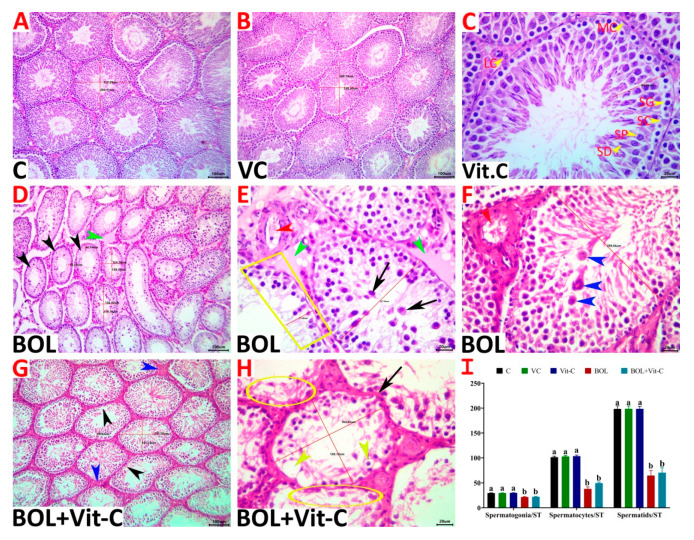
(**A**–**H**) Representative photomicrograph of H&E stained testicular tissue sections showing normal histological picture in control (**A**), vehicle control (VC) of sesame oil (**B**) and vitamin C (Vit-C) treated (**C**), treated rats (SG, spermatogonial cell; SP, spermatocyte; SD, spermatid; SC, Sertoli cell; LC, Leydig cell; MC, myoid cell). The boldenone (BOL)- treated group showed tubular degeneration/atrophy represented by increased numbers of ST/image, maturation arrest, germ cell depletion (trapezium), necrosis (black arrowheads), desquamated premature germ cells (black arrows), spermatid giant cells (blue arrowheads), and interstitial edema (green arrowheads), and congestion (red arrowheads) (**D**–**F**). The Vit-C-BOL-treated group showed vacuolated germ cells (yellow arrowheads), germ cell depletion (black arrowheads), germ cell necrosis (yellow ellipses), redundant basement membrane (black arrow), and increased interstitial tissue elements (blue arrowheads) (**G**, **H**). Scale bar is 100 microns for (**A**,**B**,**D**,**E**), and 20 microns for **C**. (I) Changes in numbers of germ cells in different experimental groups. Data are expressed as the mean ± SE (*n* = 8 replicates). Columns carrying different superscripts (a,b) are significantly different (One-way ANOVA) (*p* < 0.001).

Grossly, the testes of all animals showed no pathological lesions and were apparently normal. Still, those of the BOL and Vit-C+BOL-treated animals showed a slight reduction in the sizes and weights compared to the control. Microscopically, normal histological pictures (STs lined by pyramid-shaped Sertoli cells supporting successive populations of maturing germinal epithelium and interstitial connective tissue harbors Leydig cells, fibroblasts, and myoid cells) were seen in the testes of the control, sesame oil, and Vit- C-treated rats (Figure 3A–C).

The BOL-treated animals’ testes showed the picture of tubular degeneration/atrophy where maturation arrest accompanied by noticeable degenerative and necrotic alterations involved the germinal epithelium, particularly the spermatocytes and spermatid stages, occasionally associated with very mild circulatory and inflammatory changes in the interstitial tissue were seen in most specimens. A large proportion of STs exhibited Sertoli cell cytoplasmic vacuolation, disorganized germ cells, vacuolated germ cells, few germ cells with eosinophilic cytoplasm, condensed nuclei, exfoliated germ cells, partial germ cell loss, and giant cell formation. These changes produced a significant drop in the numbers of STs/image, the diameters of STs, germinal epithelium/STs heights, and the numbers of Sertoli cells, spermatogonia, spermatocytes, and spermatids/STs (Figure 3D–F). A few proportions of STs showed complete loss of their germ cells and redundant basement membranes. A non-significant reduction in the Leydig cells/intertubular numbers associated with interstitial edema and increased connective tissue elements was evident. The reproprotective effects of Vit-C supplementation were average, where it neither maintained the normal testicular morphology nor prevented most of the BOL-induced testicular damage. Still, the degenerative and necrotic alterations in the testicular tissues of Vit-C+BOL-treated animals were milder and less frequent than the BOL-treated animals (Figure 3G,H). The testicular morphometric quantitative assessment and the lesion scoring in all experimental groups were presented in Table 3 and Figure 3I).

### 3.7. Immunohistochemical Assessment of AR

**Figure 4:**
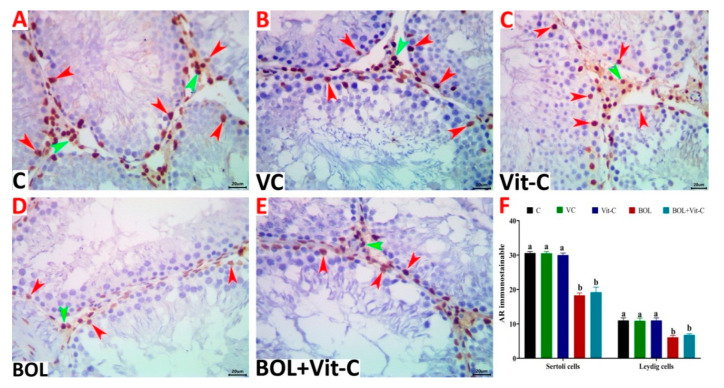
(**A**–**E**) Representative photomicrograph of testicular tissue sections of androgen receptor (AR) immunoexpression showing a marked decrease in the numbers of AR immunostainable Sertoli (red arrowheads) and Leydig cells (green arrowheads) in control (**A**), vehicle control (VC) of sesame oil (**B**), vitamin C (Vit-C) treated (**C**), boldenone (BOL) treated (**D**), and Vit-C+BOL-treated rats (**E**). The scale bar is 20 microns. (**F**) Changes in numbers of Sertoli and Leydig cells in different experimental groups. Data are expressed as the mean ± SE (*n* = 8 replicates). Columns carrying different superscripts (a,b) are significantly different (one-way ANOVA) (*p* < 0.001).

Image analysis indicated that the numbers of AR immunostainable Sertoli cells (18.30 ± 0.70) and Leydig cells (6.10 ± 0.53) in the testicular tissue sections of the BOL-treated animals were significantly (*p* < 0.001), diminished relative to the control (Sertoli cells, 30.60 ± 0.45; Leydig cells, 11.00 ± 0.76), vehicle control (Sertoli cells, 30.50 ± 0.52; Leydig cells, 10.90 ± 0.82), and Vit C-treated (Sertoli cells, 30.50 ± 0.61; Leydig cells, 11.00 ± 0.76) rats. Supplementation with Vit-C had no significant effect on the numbers of both cells (Sertoli cells, 19.20±1.45; Leydig cells, 6.80 ± 0.33) in the Vit-C+BOL-treated animals compared to the BOL-treated ones. The numbers of AR immunostainable Sertoli and Leydig cells were shown in Figure 4A–F.

## 4. Discussion

The present study demonstrated that 8 weeks intramuscular injection of male Wistar rats with BOL at the dose 5 mg/kg body weight (once/week) resulted in an increase in final body weight, body gain, abnormal sperms, serum testosterone, ACP, SDH, testicular MDA. However, decreased gonadosomatic index, male fertility indices (sperm count and motility), testicular testosterone, serum LH, FSH, and E2, and oxidative status indicators in testicular tissues were evident. Additionally, BOL-treated rats showed various pathological perturbations in the testicular tissues like depletion and complete loss of germ cells together with interstitial leukocytic infiltration, edema, and congestion. Besides, a significant reduction in the AR numbers immunostainable Sertoli cells and Leydig cells was recorded. In contrast, BOL’s concurrent treatment with Vit-C significantly recovered BOL induced sperm abnormalities, reduced sperm motility, testicular enzyme leakage, and oxidative damage but not hormonal disturbances nor AR down-regulation.

The BOL associated increased final body weight and body gain in the current study is consistent with Saleh and Waded [45]. This increase may be attributed to the holding of body nitrogen, water, potassium, sodium, and calcium ions [10]. BOL acts upon the AR in anabolic-responsive tissues, stimulating the body tissue building events due to the amplified protein synthesis. In addition, cell protein biosynthesis could be indirectly increased by anabolic hormones via stimulating the growth hormone and insulin-like growth factor release [46]. The antiglucocorticoid effects of TES and AASs are mediated through TES occupation of cortisol receptors (which have a special affinity with TES) and produce an anti-catabolic consequence [47]. Notably, BOL+Vit-C co-treated rats showed the maximum rise in body weight gain that could be related to the antioxidant activities of Vit-C, which could effectively enhance growth [48]. The positive correlation between natural products’ antioxidant activity and enhanced growth has been earlier reported in various animal species [25,49,50].

On the other hand, a significant reduction of gonadosomtic index was evident in BOL injected rats, which could be attributed to declined TES production. Similarly, some earlier reports confirmed BOL’s detrimental effect on spermatogenesis and testis size, concomitant with a testis weight reduction and developing germ cell number [10,11,51].

The significant decline in the motility and count of sperms with high sperm abnormalities in BOL treated group in our study is in accordance with Oda and El-Ashmawy [11]. In fertility clinics, AAS users have demonstrated azoosperms or oligosperma and sperm dysmorphies and dysmotility [52]. These findings could be attributed to inadequate intratesticular TES concentration, necessary for spermatogenesis [53]. Reduction of the sperm count, an increase of dead and abnormal sperm, and an increase in free radical formation, leading to germ cell apoptosis and male infertility was reported following Equigan injection in rats [54]. Additionally, the high number of abnormal sperm is an indication of the cell’s distorted structural constituents. This may have contributed to observed decreases in sperm motility provided the interrupted mitochondrial production of energy both caused by ROS generation [55].

On the other hand, Vit-C supplementation restored BOL adverse effects on epididymal sperm concentration and improved semen quality. Correspondingly, Vit-C has been reported to augment sperm motility, guard spermatogenesis, and plays a vital role in semen integrity and fertility [27,28]. The observed effects of Vit-C upon sperm motility are linked to both antioxidant and non-antioxidant, the cellular enzyme activity of the vitamin. Vit-C is essential to the production of sperm DNA content by being a coenzyme in DNA methylation. The role of healthy DNA is a healthy spermatozoon [56]. It is worth noting that the motility of sperm depends much on mitochondrial quantities and qualities in the tailpiece. In particular, it was observed that Vit-C acts as a coenzyme to reduce the oxidized components of Cu^2+^ and α-ketoglutarate-containing Fe^2+^ in the body and for kinase inhibitor [57]. This Vit-C behavior may, in one way or another, have led to the measurable result in sperm motility.

TES, the male sex hormone secreted from the testis, gives a good idea about the testis’ functional status [58]. TES is controlled through LH released from the anterior pituitary gland and plays a critical role in the final maturing of sperm. At the same time, FSH is essential for maintaining the testis gametogenic function [59]. Our results showed a potentially damaging effect of BOL injection on the testis as indicated by decrease testicular TES concentration and histopathological examination of testicular tissue. Besides, this experiment’s findings showed a substantial decrease in serum LH and FSH in rats injected with BOL. The significant reduction in TES following BOL’s exogenous administration may be attributed to inhibiting the gonadotropin-releasing hormone (GnRH) secretion from the hypothalamus and LH from the pituitary gland [15]. BOL-receptor complex had negative feedback on the hypothalamus and pituitary-gonadal axis and decreased the LH and FSH levels.

LH is controlled by GnRH, released by the hypothalamus under normal conditions. LH interacts with the Leydig cell receptors to generate TES that is then transferred to the testis and accessory reproductive organs to control tissue maintenance and growth. After exogenous AAS administration, the high androgen level triggers the LH release from the pituitary gland, which, in turn, contributes to the endogenous TES suppression [60]. AAS use dampens GnRH release either indirect effect on the pituitary gland or by blocking the hypothalamic GnRH production [52]. Tatem et al. [61] stated that Sertoli cells are unable to support spermatogenesis without adequate stimulation from FSH. At the same time, sub-par LH leads to a lower release of Leydig cell endogenous TES. However, our findings reported a significant increase in serum TES in BOL treated group compared to control groups. These findings agree with Ishak and Omar [62] and could be due to a constant supply of TES into the blood, affording a stable level of TES. In contrast, Oda and El-Ashmawy [11] reported a significant reduction in serum TES level in New Zealand mature male rabbits treated with BOL. The decrease or increase in serum TES concentrations in animals receiving AAS depends on the exposure duration, dose, and AAS type [63].

TES can be aromatized to E2 in various extra glandular tissues, a pathway that accounts for most of the estrogenic synthesis in men and postmenopausal women [64]. The current study revealed a substantial reduction in serum E2 in BOL treated group compared to the control group. BOL is TES with an extra double bond with a low rate of aromatization (about 50% of TES). In this case, it reduces the molecule’s affinity to bind to an enzyme called estrogen synthase [65]. E2 has been known to prevent the release of GnRH in men through action in the hypothalamus and pituitary [66]. The E2-TES or GnRH relationship was nonlinear. More and more E2 has less and more influence on LH, FSH, and TES circulating levels. Different concentrations of peripheral E2 in the male physiological limit resulted in low-normal TES levels to the high-normal range. The degree of androgen aromatization can differ among tissues. Also, peripheral E2 levels could not generally indicate estrogen exposure in the pituitary or hypothalamus levels. The serum concentrations of TES and E2 hormones disagreed with Ghazy and Elballal [67], where BOL injection decreased serum TES and increased E2 concentrations in rabbits.

Our findings revealed a substantial increase in ACP and SDH in the BOL treated group’s serum relative to the control group. The increased ACP and SDH enzymes activity could be linked to compromised integrity of the testes comprising severe disorganization of the germinal cell, degeneration, and spermatogenic elements destruction resulting in leakage of these enzymes into the bloodstream. Co-treatment of BOL with Vit-C significantly suppressed the BOL induced increment of serum testicular enzymes. Consistent with the previous studies by Anvari et al. [68], which reported that Vit-C is necessary for the functional and structural integrity of reproductive organs that are androgen-dependent.

Our results revealed a substantial decrease in SOD, CAT, and GSH but a significant rise in MDA levels indicating that BOL treatment induces oxidative stress in rat testis. Similarly, Ali et al. [69] verified the decrease of SOD and GSH and increased MDA in muscle tissues in New Zealand rabbits after BOL injection. Furthermore, Ibrahim and Said [70] reported that significantly inductive lipid peroxidation and fragmentation of DNA, as well as complete antioxidant capacity (TAC) and CAT activity inhibition, occurred with intramuscular BOL injecting in testis and kidney tissue. Also, Pinheiro, et al. [71] reported that AAS usage increases the peroxidation of lipids without raising the total antioxidants.

Vit C is essential in preventing oxidative damage to the sperm and steroid cells of the Leydig cells and in the sperm chamber [72]. In the current study, oral co-treatment of rats with Vit-C and BOL significantly increases the testicular CAT, SOD, and GSH level but decreases the testis’s MDA content. These findings demonstrate how the Vit-C protects against oxidative stress triggered by BOL. Vit-C is an excellent electron source, giving an electron to free radicals such as superoxides and hydroxyls radicals, which reduces their reactivity [73]. Our findings are following Kini et al. [74], who demonstrated a significant rise in the testicular GSH and SOD level but a decrease in testicular MDA level after pretreatment of rats with Vit-C before cadmium chloride exposure.

Androgen activity is mediated by AR transcriptional activation in the testis, as in other tissues. TES is selectively attached to AR in Sertoli cells, and receptor activation will trigger and sustain the spermatogenic process and prevent the apoptosis of germ cells [75]. In the testis and epithelial and interstitial cells of the epididymis, immunosustainable AR was observed in Sertoli cells, peritubular myoid cells, and Leydig cells [76]. In the current work, intramuscular injection of male rats with BOL resulted in the reduction of testicular TES, which caused a significant reduction in the numbers of STs/image, the diameters of STs, heights of germinal epithelium/STs, and the Sertoli cells numbers, spermatogonia, spermatocytes, and spermatids/STs with a few proportions of STs showed complete loss of their germ cells and redundant basement membranes. Birgner et al. [77] found that AR loss from Sertoli cells would result in incomplete meiosis and collapse of a sperm cell to haploid round spermatids. The number of positive nuclei inside ST’s borders was identified in the present study for immunoexpression of AR. Furthermore, BOL treatment has been found to decrease the number of Sertoli AR cells, thereby explaining the observed maturing disturbances and testicular atrophy. Sertoli cells play an essential part in the organization and function of the somatic cell lineages and in determining the testis structure. They often endorse a small number of germ cells, thereby deciding the adult’s sperm capability [78]. The present findings indicate the BOL-reduced cell number of Sertoli due to Sertoli’s structural response to deprivation from TES [79]. The decrease in the cell number of Sertoli in BOL treated rats would have contributed to a corresponding reduction in the sperm count. Similarly, Bahey et al. [80] reported that a significant decrease in AR immunoexpression which was related mainly to TES depletion in diabetic conditions. Also, the Sertoli cells’ responses to stimulation by FSH or androgens would rely on information conveyed from neighboring germinal cells at definite stages of the seminiferous epithelium [81]. Surprisingly, Vit-C could not modify the AR altered expression due to BOL as well as the disturbed hormonal profile. These findings imply that Vit-C did not hinder the BOL depressive actions on the hypothalamus that secrete GnRH that manages pituitary gonadotrophs. Thus, we could suppose that the profertility effects of Vit-C in BOL treated rats are mainly linked to its antioxidant activities, not to its ability to raise the blood TES level. In this context, Kehinde et al. [48] demonstrated that Vit-C with its anti-oxidant activity did not inhibit the sodium benzoate depressive actions on the hypothalamus that release GnRH which controls pituitary gonadotrophes.

## 5. Conclusions

The current findings conclude that BOL could impair male fertility by several mechanisms, including evoking oxidative stress in the testis, impair GnRH and consequently TES testicular release, and downregulation of AR immunoexpression. Notably, Vit-C as a protective agent with BOL could significantly recover its oxidative damage in the testis, but not hormonal disturbances or AR down-regulation. Further studies are highly warranted to address if Vit-C could help repair the resultant testicular oxidative damage following a long duration of AAS administration. Additionally, exploring other natural products targeted AR to rescue BOL induced impaired fertility is highly needed.

## Figures and Tables

**Table 1:** Effect of boldenone (5 mg/kg bwt/once a week, eight weeks) and/or vitamin C (120 mg/kg b.wt/daily, eight weeks) treatment on body weight change, gonadosomatic index, and sperm characteristics of adult male Wister rats.

Groups	Control	Vehicle Control	Vit-C	BOL	BOL+Vit-C	*p*-Value
Initial body weight (g)	165.33 ± 2.31	166.00 ± 1.76	168.75 ± 1.50	165.50 ± 1.43	168.50 ±1.50	0.465
Final body weight (g)	204.93 ^d^ ± 4.47	205.25 ^d^ ± 1.20	220.25 ^c^ ± 1.59	247.00 ^b^ ± 1.84	275.75 ^a^ ± 5.89	<0.001
Body weight gain (g)	39.60 ^c^ ± 4.86	39.25 ^c^ ± 1.98	51.50 ^c^ ± 2.64	81.50 ^b^ ± 2.50	107.25 ^a^ ± 7.15	<0.001
Gonadosomatic index (%)	1.00^a^ ± 0.05	0.89^ab^ ± 0.05	1.01 ^a^ ± 0.05	0.73^c^ ± 0.03	0.79 ^bc^ ± 0.02	<0.001
Sperm count (sp.cc/mL×125×10^4^)	37.67 ^a^ ± 1.31	38.00 ^a^ ± 1.08	40.00 ^a^ ± 1.22	16.00^b^ ± 1.47	17.33^b^± 1.25	<0.001
Sperm motility (%)	87.00 ^a^ ± 1.08	86.33 ^a^ ± 1.43	89.33 ^a^ ± 2.09	60.00 ^c^ ± 2.04	79.67 ^b^ ± 1.84	<0.001
Sperm abnormalities (%)	17.24 ^c^ ± 0.32	17.26 ^c^ ± 0.27	15.58 ^c^ ± 0.50	40.82^a^ ± 0.47	24.26 ^b^ ± 1.31	<0.001

BOL: boldenone and Vit-C: vitamin C. A one-way analysis of variance (ANOVA) followed by Duncan’s Multiple Range test was used for statistical analysis. Means within the same row carrying different superscripts (^a–d^) are significantly different. The values shown are means ± SE. *n* = 8.

**Table 2:** Effect of boldenone (5 mg/kg bwt/once a week, eight weeks) and/or vitamin C (120 mg/kg b.wt/daily, eight weeks) treatment on hormonal variables and testicular enzymes of adult male Wister rats.

Groups	Control	Vehicle Control	Vit-C	BOL	BOL+Vit-C	*p*-Value
Serum TES (ng/mL)	2.87 ^b^ ± 0.31	3.65 ^b^ ± 0.23	3.88 ^b^ ± 0.75	7.19 ^a^ ± 0.08	6.12 ^a^ ± 0.79	<0.001
Testicular TES (ng/g tissue)	406.40^a^ ± 9.86	400.00^a^ ± 13.76	400.00 ^a^ ± 14.71	147.20 ^b^ ± 5.99	171.20^b^ ± 8.16	<0.001
LH (mIU/mL)	3.43 ^a^ ± 0.08	3.51 ^a^ ± 0.06	3.53 ^a^ ± 0.06	2.28 ^b^ ± 0.32	2.67 ^b^ ± 0.15	<0.001
FSH (ng/mL)	8.31 ^a^ ± 0.39	8.35 ^a^ ± 0.23	8.45 ^a^ ± 0.29	4.54 ^b^ ± 0.50	5.63 ^b^ ± 0.38	<0.001
E2 (pg/mL)	59.77 ^a^ ± 3.20	58.10 ^a^ ± 3.34	57.67 ^a^ ± 3.14	28.20 ^b^ ± 1.28	35.13^b^ ± 1.82	<0.001
ACP (U/L)	15.67 ^c^ ± 1.65	16.67^c^ ± 1.43	16.33 ^c^ ± 1.25	47.33 ^a^ ± 2.72	25.67 ^b^ ± 2.46	<0.001
SDH (ng/mL)	1.38 ^c^ ± 0.07	1.39 ^c^ ± 0.19	1.38 ^c^ ± 0.10	5.00 ^a^ ± 0.14	2.36 ^b^ ± 0.26	<0.001

BOL: boldenone; Vit-C: vitamin C; TES: testosterone; LH: luteinizing hormone; FSH: follicle-stimulating hormone; E2: estradiol; ACP: acid phosphatase; SDH: sorbitol dehydrogenase. A one-way analysis of variance (ANOVA) followed by Duncan’s Multiple Range Test was used for statistical analysis. Means within the same row carrying different superscripts (^a–d^) are significantly different. The values shown are the means ± SE. *n* = 8.

**Table 3:** Lesion scoring in the testicular tissues of rats in response to boldenone (5 mg/kg bwt/once a week, 8 weeks) and/or vitamin C (120 mg/kg b.wt/daily, 8 weeks) treatment.

Lesion	Control	Vehicle Control	Vit-C	BOL	Vit-C+BOL	*p*-Value
ST/10X	15.40 ^b^ ± 0.69	15.90 ^b^ ± 0.67	15.50 ^b^ ± 0.72	24.60 ^a^ ± 0.70	23.00 ^a^ ± 0.61	<0.001
Diameter of ST	275.09 ^a^ ± 6.22	275.58 ^a^ ± 6.07	279.05 ^a^ ± 5.80	175.23 ^b^ ± 11.33	184.41 ^b^ ± 10.81	<0.001
Height of germinal epithelium/ST	83.18 ^a^ ± 1.68	83.54 ^a^ ± 1.58	83.81 ^a^ ± 2.09	44.04 ^b^ ± 3.08	46.26 ^b^ ± 2.26	<0.001
ST with vacuolated germinal epithelium	0.00 ^c^ ± 0.00	0.00 ^c^ ± 0.00	0.00 ^c^ ± 0.00	5.66 ^a^ ± 0.73	4.05 ^b^± 0.23	<0.001
ST with detached germinal epithelium	0.12 ^c^ ± 0.12	0.13 ^c^ ± 0.13	0.13 ^c^ ± 0.13	18.10 ^a^ ± 1.22	14.54 ^b^ ± 1.96	<0.001
STs with depleted germ cells	0.00 ^c^ ± 0.00	0.00 ^c^ ± 0.00	0.00 ^c^ ± 0.00	64.89 ^a^ ± 3.73	57.86 ^b^ ± 3.60	<0.001
ST with necrotic germinal epithelium	0.00 ^c^ ± 0.00	0.00 ^c^ ± 0.00	0.00 ^c^ ± 0.00	58.65 ^a^ ± 2.39	50.07 ^b^ ± 1.67	<0.001
STs with complete loss of germ cells	0.00 ^c^ ± 0.00	0.00 ^c^ ± 0.00	0.00 ^c^ ± 0.00	2.04 ^a^ ± 0.35	1.22 ^b^ ± 0.18	<0.001
STs with redundant basement membranes	0.00 ^b^ ± 0.00	0.00 ^b^ ± 0.00	0.00 ^b^ ± 0.00	0.99 ^a^ ± 0.30	0.45 ^b^ ± 0.25	<0.001
Spermatid retention	0.00 ^c^ ± 0.00	0.00 ^c^ ± 0.00	0.00 ^c^ ± 0.00	0.65 ^a^ ± 0.02	0.32 ^b^ ± 0.01	<0.001
Interstitial leukocytic infiltration	0.00 ^c^ ± 0.00	0.00 ^c^ ± 0.00	0.00 ^c^ ± 0.00	4.10 ^a^ ± 0.80	2.05 ^b^ ± 0.40	<0.001
Interstitial edema	0.00 ^c^ ± 0.00	0.00 ^c^ ± 0.00	0.00 ^c^ ± 0.00	8.00 ^a^ ± 0.73	4.00 ^b^ ± 0.37	<0.001
Interstitial congestion	0.00 ^b^ ± 0.00	0.00 ^b^ ± 0.00	0.00 ^b^ ± 0.00	10.00 ^a^ ± 3.33	4.00 ^b^ ± 2.67	0.001

BOL: boldenone and Vit-C: vitamin C.Values are mean ± SE for *n* = 5 samples/group. Means within the same row (in each parameter) carrying different superscripts (a, b, and c) are significantly different at *p* < 0.05 groups. Data are expressed as the mean ± SE (*n* = 8 replicates). Columns carrying different superscripts (^a–c^) are significantly different (one-way ANOVA).
